# Top-down control and early multisensory processes: chicken vs. egg

**DOI:** 10.3389/fnint.2015.00017

**Published:** 2015-03-03

**Authors:** Rosanna De Meo, Micah M. Murray, Stephanie Clarke, Pawel J. Matusz

**Affiliations:** ^1^Neuropsychology and Neurorehabilitation Service, Centre Hospitalier Universitaire Vaudois and University of LausanneLausanne, Switzerland; ^2^Electroencephalography Brain Mapping Core, Center for Biomedical Imaging (CIBM)Lausanne and Geneva, Switzerland; ^3^The Laboratory for Investigative Neurophysiology, Neuropsychology and Neurorehabilitation Service and Department of Radiology, Centre Hospitalier Universitaire Vaudois and University of LausanneLausanne, Switzerland; ^4^Faculty in Wroclaw, University of Social Sciences and HumanitiesWroclaw, Poland; ^5^Attention, Brain and Cognitive Development Group, Department of Experimental Psychology, University of OxfordOxford, UK

**Keywords:** attention, control processes, top-down control, bottom-up, multisensory, EEG/ERP, crossmodal

Traditional views contend that behaviorally-relevant multisensory interactions occur relatively late during stimulus processing and subsequently to influences of (top-down) attentional control. In contrast, work from the last 15 years shows that information from different senses is integrated in the brain also during the initial 100 ms after stimulus onset and within low-level cortices. Critically, many of these early-latency multisensory interactions (hereafter *eMSI*) directly impact behavior. The prevalence of eMSI substantially advances our understanding of how unified perception and goal-related behavior emerge. However, it also raises important questions about the dependency of the eMSI on top-down, goal-based attentional control mechanisms that bias information processing toward task-relevant objects (hereafter *top-down control*). To date, this dependency remains controversial, because eMSI can occur independently of top-down control, making it plausible for (some) multisensory processes to directly shape perception and behavior. In other words, the former is not necessary for these early effects to occur and to link them with perception (see Figure 1A). This issue epitomizes the fundamental question regarding direct links between sensation, perception, and behavior (*direct perception*), and also extends it in a crucial way to incorporate the multisensory nature of everyday experience. At the same time, the emerging framework must strive to also incorporate the variety of higher-order control mechanisms that likely influence multisensory stimulus responses but which are not based on task-relevance. This article presents a critical perspective about the importance of top-down control for eMSI: In other words, who is controlling whom?

**Figure 1:**
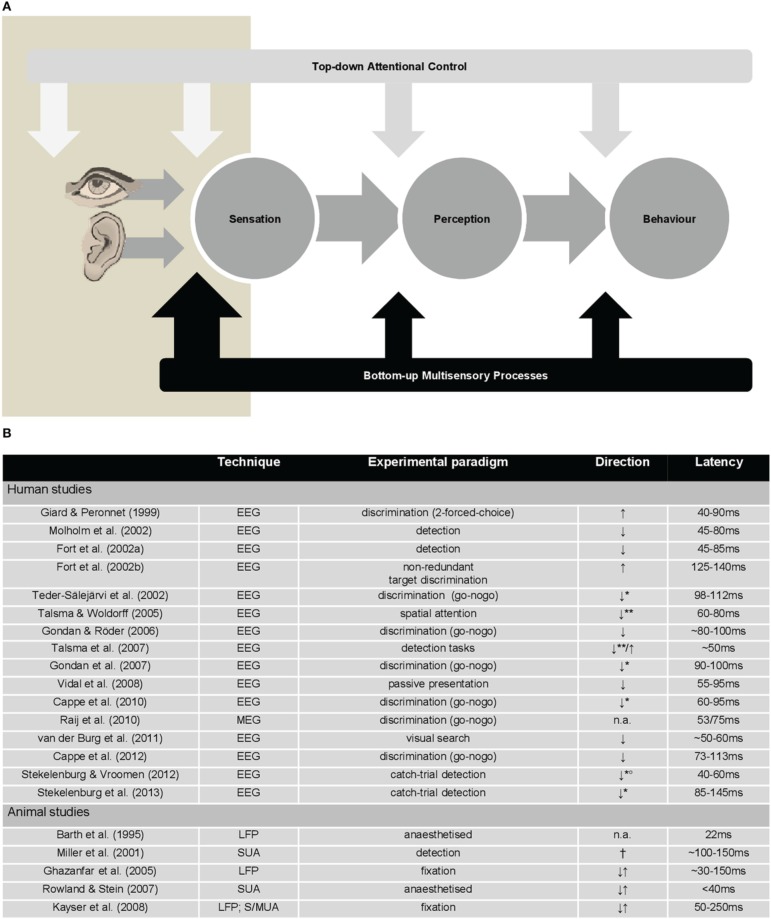
**(A)** Depiction of manners in which top-down attentional control and bottom-up multisensory processes may influence direct perception in multisensory contexts. In this model, the bottom-up multisensory processes that occur early in time (eMSI; beige box) have direct effects on perception and behavior (large black arrow). In turn, top-down attentional control mechanisms, which are typically posited to exert effects at multiple pre-stimulus and post-stimulus stages, do not seem to do so in some multisensory contexts (white arrows). **(B)** Table summarizing principal findings on eMSI from human EEG/MEG studies and animal electrophysiological studies. Note: EEG, electroencephalography; MEG, magnetoencephalography; LFP, local field potentials; SUA, single-unit activity; MUA, multi-unit activity; ↑, sub-additive responses; ↓, super-additive responses; ^**^, responses elicited by irrelevant-but-attended multisensory stimuli; ^**^, responses elicited by unattended multisensory stimuli; °, eMSI found only for spatially congruent audiovisual stimuli; †, eMSI found on the response latency, but not on the response amplitude; n.a., data not available.

## The ubiquity of eMSI

For the purposes of this article we focus exclusively on auditory-visual interactions and define *eMSI* as those multisensory processes that occur within the first 100 ms post-stimulus onset (but see (Giard and Peronnet, 1999); Giard and Peronnet, who qualified effects <200 ms as early-latency). This definition is in keeping with influential models of visual perception and attentional selection, positing that top-down and recursive inputs manifest after the initial 100 ms of stimulus-driven brain activity, which is believed to be sensory-perceptual and bottom-up in nature (e.g., Luck et al., 1997; Lamme and Roelfsema, 2000). It is likewise important to distinguish between *integration* effects, which are responses elicited by a combination of inputs to different senses, and *cross-modal* effects, which refer to influences of inputs to one sense on activity associated with another sense (e.g., Stein et al., 2010).

The typical perceptual outcome of multisensory integration is that stimulus processing is facilitated (as shown by faster and/or more accurate responses) in contexts where inputs to different senses are carrying similar (*redundant*) information and are presented close in time. This behavioral facilitation is typically accompanied by brain responses to multisensory stimuli that diverge from the summed brain responses to the constituent unisensory signals (*nonlinear responses*; Figure 1B). Given the growing evidence for links between the brain and the behavioral responses (reviewed in Murray et al., 2012), one mechanism may be that the temporal co-occurrence of multisensory information lowers the threshold for neural activity that in turn drives perception and action (e.g., Rowland and Stein, 2007).

Based on the extant literature, we argue that these particular multisensory processes, which are reflected by eMSI, are stimulus-driven, *bottom-up* in nature and affect perception and behavior in a direct manner and largely independently of top-down control (Figure 1A). The idea of a variety, or even a range of multisensory processes, where some are “automatic” while others dependent on one's current behavioral goals, has until now been systematically investigated mainly in the context of attentional selection of objects in space, rather than their perception *per se* (e.g., Matusz and Eimer, 2011, 2013; Matusz et al., 2015; Talsma and Woldorff, 2005; but see Murray et al., 2004; Soto-Faraco et al., 2004; Tiippana et al., 2004; Alsius et al., 2005, 2007; Thelen et al., 2012, 2014; Matusz et al., in press). However, control processes are likely to be important for both cognitive functions (e.g., Gunseli et al., 2014); this should hold for both unisensory and multisensory processes, and bottom-up and top-down processes alike (i.e., multisensory processes are not mechanistically “special”; van Atteveldt et al., 2014).

It is difficult to argue with the idea that early responses are a hallmark of bottom-up multisensory processes in the service of perception, if one considers how ubiquitous and context-independent they are in both humans and in the animal models (see Figure 1B; reviewed in Murray et al., 2012; Kajikawa et al., 2012). The eMSI in local field potentials as well as spiking activity have been measured in the primary and secondary auditory fields of fixating monkeys (Ghazanfar et al., 2005; Kayser et al., 2008; see also Lakatos et al., 2008; Wang et al., 2008 for cross-modal effects). Importantly, these eMSI occurred for both ethological objects (conspecific communication signals) and simple audiovisual stimuli, though modulated according to bottom-up stimulus salience and neural efficacy. Moreover, non-linear interactions mirroring the behavioral gains in stimulus detection have been recorded in single neurons in the area 4 of the monkey motor cortex within 100–150 ms post-stimulus (Miller et al., 2001).

Electroencephalography and magnetoencephalography (EEG/MEG) studies in humans have likewise demonstrated eMSI across a variety of tasks, ranging from simple detection (Fort et al., 2002a; Molholm et al., 2002) and discrimination (Giard and Peronnet, 1999; Fort et al., 2002b; Teder-Sälejärvi et al., 2002; Gondan and Röder, 2006; Gondan et al., 2007; Raij et al., 2010; Cappe et al., 2010, 2012; Stekelenburg and Vroomen, 2012; Stekelenburg et al., 2013) tasks to multi-stimulus/ multi-stream paradigms necessitating selection (Talsma and Woldorff, 2005; Talsma et al., 2007; van der Burg et al., 2008, 2011). Importantly, eMSI were observed irrespective of whether the multisensory stimuli were targets (e.g., Giard and Peronnet, 1999; Pérez-Bellido et al., 2013), attended but task-irrelevant stimuli (e.g., Cappe et al., 2010) or were presented passively (Vidal et al., 2008). As will be detailed below, data from brain stimulation studies allow causal inference regarding behavioral consequences of eMSI (see below).

The interpretability of the eMSI in terms of bottom-up vs. top-down mechanisms critically depends on their localization. Despite the ubiquity of the eMSI in extant EEG/MEG studies, only few have applied the requisite signal analysis and source reconstruction methods. Localization results support the predominant role of low-level cortices in the eMSI (Cappe et al., 2010; Raij et al., 2010). While the localization of the eMSI to low-level cortices could be taken as evidence for their strictly bottom-up nature, their latency at ~50–100 ms is sufficiently “late” to provide ample opportunity for recursive processing (Musacchia and Schroeder, 2009; also Moran and Reilly, 2006 for modeling results). This may involve top-down modulation or the extraction and disambiguation of stimulus features (Lamme and Roelfsema, 2000). Thus, care is warranted in regarding all eMSI as indicative of bottom-up multisensory integration. For example, *the pip and pop* effect (van der Burg et al., 2008) triggers eMSI-like responses, but only in the case of targets, not distractors (van der Burg et al., 2011). Thus, dependency of the eMSI on top-down control can be assessed only by analyzing studies where the latter is directly manipulated^1^.

## The chicken: top-down control and its limited role in eMSI

The strongest evidence for the dependence of eMSI on top-down control comes from studies where attended and unattended multisensory stimuli were directly compared (e.g., Alsius et al., 2005, 2007; Talsma and Woldorff, 2005; Talsma et al., 2007). However, the literature seems prone to misconstruing the full breadth of the results. In one study participants detected infrequent targets in one of two central streams of rapidly presented alphanumeric symbols or combinations of beeps and flashes (Talsma et al., 2007). When attended, audiovisual stimuli triggered early enhanced (*super-additive*) nonlinear responses. But, when the competing stream was attended, these nonlinear interactions changed polarity, becoming suppressed (*sub-additive*). One interpretation of these results is that top-down control regulates multisensory integration, from its magnitude and quality to its very presence (Koelewijn et al., 2010). We believe this viewpoint should perhaps be more nuanced. The top-down control manipulations modulated the eMSI, but did not eliminate them. Additionally, the eMSI were observed despite the paradigm manipulating in fact *multiple* top-down mechanisms (inter-modal, but also spatial, feature-based, and object-based). While further research is required to fully characterize the mechanistic underpinnings of super- vs. sub-additive interactions, the results of this study are in line with the importance of top-down control processes revealed by unisensory studies, wherein responses to stimuli are enhanced according to the task-relevance of their location, features or identity (reviewed in Nobre and Kastner, 2013). Talsma et al. (2007) was the first to demonstrate the pivotal role of the task-relevance of multisensory pairings for the *quality* of the eMSI they trigger. However, the *presence* of the eMSI in this study was independent of task-relevance, though some evidence would suggest that the eMSI are preferentially observed in *unattended* contexts (Table 2 in Talsma and Woldorff, 2005). This latter evidence is in line with the eMSI being a hallmark of stimulus-driven processing.

It is difficult to ignore that in these few studies, where top-down control mechanisms were directly manipulated, the eMSI were sub-additive in nature. What is striking is that this is precisely the direction of effects reported in the literature irrespective of whether responses to targets, non-targets or passively presented stimuli are considered (Figure 1B). Historically, sub-additive effects were dismissed as confounds related to common activity across both unisensory and multisensory conditions. More recently, they have been increasingly recognized as a canonical mechanism that can convey information particularly efficiently (Kayser et al., 2009; Altieri et al., in press; reviewed in Stevenson et al., 2014). The issue of the quantification of the eMSI is further complicated by the fact that the overwhelming majority of the human EEG studies have used relative, reference-dependent measures of amplitude (cf., Murray et al., 2008).

## The egg: eMSI as a bottom-up phenomenon

Several independent lines of research across various species provide converging evidence for the bottom-up nature of the eMSI. On the one hand, there are reports of eMSI in anesthetized animals (e.g., rats, Barth et al., 1995; cats, Rowland and Stein, 2007; see also reviews in Sarko et al., 2012; Rowland and Stein, 2014), where top-down modulations are blocked^2^. On the other hand, sounds have been shown to enhance the excitability of low-level visual cortices, as measured via phosphene perception. Several aspects of this effect demonstrated by TMS studies in humans support the bottom-up nature of the eMSI and the causal links between eMSI and behavior (Romei et al., 2007, 2009, 2013; Spierer et al., 2013).

First, it is modulated by low-level sound features, with greater excitability increases observed for narrowband and higher pitch sounds. Visual cortex excitability is furthermore enhanced selectively by structured approaching (looming) sounds versus stationary or receding sounds as well as non-structured white-noise versions of these sounds. Second, the effect is delimited in time, occurring when sounds precede the TMS by 30–150 ms, in correspondence with the eMSI identified using EEG/MEG. Third, the sound-induced enhancements of visual cortex excitability transpire before subjects can explicitly differentiate between the sounds, i.e., at pre-perceptual processing stages. Relatedly, increases in the occipital excitability occur with sounds that themselves fail to elicit startle responses, arguing against an alerting explanation. Fourth, evidence against a top-down account of these effects comes from studies demonstrating that individuals' attentional preference (as independently measured in an auditory-visual divided attention task) affect late, but not early, stages of the excitability changes.

Finally, the TMS-driven visual cortex activity is behaviorally relevant. Occipital TMS delivered 60–90 ms post-stimulus has opposing effects of roughly equal magnitude (~15 ms) on reaction times to unisensory auditory and visual stimuli (speeding and slowing, respectively) and has no measurable effect on reaction times to simultaneous auditory-visual multisensory stimuli. Critically, the response speed facilitation obtained from the combination of occipital TMS and an external auditory stimulus was as great as and correlated with that obtained from presenting participants with genuine multisensory stimuli. The TMS-induced cross-modal effects seem to emulate those observed with multisensory stimuli.

## Conclusions and future directions

We demonstrated that the eMSI are robust phenomena, observable across species, experimental paradigms and measures of neural activity (Figure 1B). To refer more explicitly to the Research Topic of this issue, we subscribe to a view of multiple multisensory processes: The eMSI are a hallmark of bottom-up multisensory processes that facilitate perception and behavior directly, independently of top-down control (Figure 1A).

We focused here exclusively on stimulus-locked brain activity. Thus, temporal dynamics complement the understanding of the interplay between bottom-up and top-down mental processes as hitherto provided from the vantage-point of brain oscillations, which assay both intra-population excitability as well as inter-population communication (Thut et al., 2012; van Atteveldt et al., 2014).

A critical next step will be the detailed mechanistic characterization of the eMSI. The sub-additive archetype of the eMSI goes together with the evidence from unisensory research linking reduced responses with more efficient and information-rich processing akin to the repetition suppression phenomena and the predictive coding accounts (e.g., Grill-Spector et al., 2006; Summerfield and Egner, 2009). When and why do top-down control processes flip the sub-additive eMSI to become super-additive? If top-down control affects the nature, rather than the presence, of multisensory processes, then what are the consequences for our understanding of perception? Paradoxically, while the eMSI are on the one hand upturning somewhat dogmatic views on the brain functional organization, they simultaneously are entrenching a classic model of perceptual processing positing direct links between sensation, perception, and behavior. An accurate picture of the nature of perceptual processes is thus provided by studying them in naturalistic, multisensory contexts and where the task demands dynamically vary.

## Author contributions

All authors have contributed to all aspects of this work. All authors have approved the final version of the manuscript and agreed to be held accountable for all aspects of the work.

### Conflict of interest statement

The authors declare that the research was conducted in the absence of any commercial or financial relationships that could be construed as a potential conflict of interest.

## Footnotes

^1^To our knowledge semantic congruence does not modulate eMSI (Fort et al., 2002b; Molholm et al., 2004; Yuval-Greenberg and Deouell, 2007).

^2^We would hasten to remind the reader that convergent anatomical input is necessary but in and of itself insufficient for eMSI as defined it in this opinion piece. It is true that the anatomic pathways/connectivities as well as their shaping by experiences are prerequisites for multisensory processes. However, the activation of these physical substrates in relation to the cascade of sensory-evoked responses must be sufficiently early so as to influence perception and behavior directly and thus be qualified as eMSI.
